# Investigating the Effect of Xylitol on *ompK36* Overexpression, Increased Meropenem Susceptibility, and Antibiofilm Activity in a Carbapenem‐Resistant Clinical Strain of *Klebsiella pneumoniae*

**DOI:** 10.1155/ijm/3754560

**Published:** 2026-05-12

**Authors:** Omid Ahlelyorof, John Flanagan, Fereshteh Jabalameli, Reza Beigverdi, Maryam Siroosi

**Affiliations:** ^1^ Department of Microbiology, School of Medicine, Tehran University of Medical Sciences, Tehran, Iran, tums.ac.ir; ^2^ Department of Precision and Molecular Medicine, Penn State College of Medicine, Hershey, Pennsylvania, USA, pennstatehershey.org; ^3^ Research Center for Antibiotic Stewardship and Antimicrobial Resistance, Tehran University of Medical Sciences, Tehran, Iran, tums.ac.ir

**Keywords:** biofilm, carbapenem, *Klebsiella pneumoniae*, meropenem, xylitol

## Abstract

Carbapenem‐resistant *Klebsiella pneumoniae* poses a significant public health threat due to limited therapeutic options. This study investigated the potential of xylitol, a sugar alcohol, to enhance meropenem activity and combat biofilms in a clinical carbapenem‐resistant *K. pneumoniae* strain (Kp5) and a standard susceptible strain (*K. pneumoniae* subsp. *pneumoniae* ATCC 13883). The synergistic activity between xylitol and meropenem in inhibiting bacterial growth was assessed using a two‐dimensional checkerboard assay. Real‐time PCR was performed to assess the effect of xylitol on the expression of the porin gene *ompK36*, a key determinant of carbapenem entry and resistance in *K. pneumoniae*. Moreover, the microtiter plate assay was used to evaluate its antibiofilm efficacy. Results showed that xylitol exhibited a synergistic effect with meropenem, leading to a significant reduction in the meropenem minimum inhibitory concentration (MIC) in both strains. Notably, the meropenem MIC for the Kp5 strain was reduced by up to 32‐fold in the presence of xylitol. Exposure to xylitol alone induced a significant upregulation of *ompK36* expression in both strains, suggesting that increased porin gene expression may enhance meropenem activity. Furthermore, xylitol demonstrated potent, concentration‐dependent antibiofilm activity, both inhibiting biofilm formation and eradicating established biofilms. This approach offers a promising strategy for repurposing a safe and readily available compound to address a critical aspect of antimicrobial resistance.

## 1. Introduction

*Klebsiella pneumoniae* is a Gram‐negative bacterium responsible for a range of nosocomial and community‐acquired infections, including pneumonia, sepsis, meningitis, and urinary tract infections [1]. The global rise in multidrug‐ and carbapenem‐resistant *K. pneumoniae* strains is a significant public health concern, largely due to inappropriate antimicrobial stewardship and widespread antibiotic exposure, particularly carbapenems [2, 3]. Carbapenems, such as meropenem, imipenem, ertapenem, and doripenem, are *β*‐lactam antibiotics with the broadest spectrum of activity compared to other *β*‐lactams [4]. Carbapenem‐resistant infections are associated with increased mortality and substantial healthcare costs across diverse economic settings [3]. Resistance in *K. pneumoniae* is attributed to multiple mechanisms, including the production of enzymes that modify or hydrolyze antibiotics and the action of efflux pumps that expel antibiotics from the cell. Furthermore, the bacterium′s ability to form biofilms presents a significant challenge, as its matrix acts as a barrier to antimicrobial agents, thereby reducing susceptibility. Another key resistance mechanism involves the reduction of antibiotic influx into the cell, often mediated by the downregulation of specific porins [1, 2]. Porins are outer membrane proteins that form water‐filled channels in the outer membrane of various bacteria to facilitate the diffusion of small molecules, including antibiotics, into the cell. OmpK35 and OmpK36, homologous to *Escherichia coli* OmpF and OmpC, respectively, are major porins in *K. pneumoniae* [5]. Notably, OmpK36 expression is upregulated under high osmotic pressure [6], and its absence is strongly linked to increased carbapenem resistance [7]. Moreover, both OmpK35 and OmpK36 contribute to quinolone susceptibility, as their loss of expression contributes to resistance [8].

Several treatment strategies exist to combat carbapenem‐resistant Enterobacteriaceae infections. For carbapenemase‐mediated resistance, *β*‐lactamase inhibitors offer a promising approach for specific carbapenemase types; however, their efficacy is limited and not universal. Clinically available *β*‐lactam‐*β*‐lactamase inhibitor combinations include ceftazidime–avibactam, meropenem–vaborbactam, and imipenem–relebactam. Colistin remains crucial for managing carbapenem‐resistant infections, despite growing resistance. Tigecycline is also a viable option for certain infections, but side effects have been reported [9]. Given the cost burdens on healthcare systems caused by these infections, novel therapies are being explored. Nanoparticle‐based antimicrobials with broad‐spectrum activity have been investigated against various bacteria, including members of the Enterobacteriaceae family. Phage therapy presents another alternative with limited side effects; however, its bacterium‐specific mechanism can be both an advantage and a limitation, and it does not prevent the emergence of resistance. Vaccine development studies for infection prevention are also ongoing [10]. Given the limitations and increasing resistance to the existing therapies, novel strategies are urgently needed. As mentioned earlier, upregulation of porins under high osmotic stress to enhance antibiotic influx represents a promising yet underexplored strategy to increase *K. pneumoniae*′s susceptibility to antibiotics.

Xylitol is a five‐carbon sugar alcohol widely used as an artificial sweetener that is well known for its dental antiplaque properties, as well as its ability to reduce or inhibit inflammatory responses [11]. It is commonly incorporated into toothpastes, mouthwashes, nasal sprays, chewing gums, and some other formulations [12]. Compared with other polyols such as sorbitol, xylitol is generally well tolerated in adults, even at oral intakes of up to 75 g/day; however, gastrointestinal effects, including bloating, have been reported in some individuals [13]. The antibacterial and antibiofilm effects of xylitol have been extensively investigated, particularly against *Pseudomonas aeruginosa*, *Staphylococcus aureus*, and *Streptococcus mutans* [14–18], whereas its effects on *K. pneumoniae* remain largely unexplored.

In this study, we focused on the outer membrane porin OmpK36, a key determinant of carbapenem uptake in *K. pneumoniae*. Its expression is regulated by osmotic stress, and downregulation of OmpK36 is associated with carbapenem resistance. We investigated the potential of xylitol, which can induce osmotic stress, to upregulate *ompK36* gene expression and enhance meropenem susceptibility in a clinical, multidrug‐ and carbapenem‐resistant *K. pneumoniae* strain. To accomplish this, we employed real‐time PCR to quantify changes in the *ompK36* gene expression. Additionally, we explored the effects of xylitol‐induced osmotic stress on biofilm formation and eradication by this strain.

## 2. Materials and Methods

### 2.1. Bacterial Strains

A multidrug‐ and carbapenem‐resistant *K. pneumoniae* strain (Kp5) was received from a hospital clinical microbiology laboratory [19], where its initial phenotypic identification had been performed. Species confirmation was achieved by PCR amplification of the *khe* gene, a specific marker for *K. pneumoniae*, using DNA extracted via the boiling method [20]. This strain served as the clinical strain in our study. Additionally, *K. pneumoniae* subsp. *pneumoniae* ATCC 13883, a porin‐expressing reference strain, was included as the standard control for all experiments. Both strains were stored at −70°C in glycerol suspensions until further use.

### 2.2. Antimicrobial Susceptibility Testing

Antibiotic susceptibility of the Kp5 strain to ceftazidime, ciprofloxacin, gentamicin, imipenem, piperacillin–tazobactam, tobramycin, and trimethoprim–sulfamethoxazole was determined using the disk diffusion method. Clear zone diameters around disks were measured in millimeters and interpreted as susceptible (*S*), intermediate (*I*), or resistant (*R*) according to Clinical and Laboratory Standards Institute (CLSI) guidelines. Disk diffusion breakpoints for Enterobacterales were applied as follows: ceftazidime (*S* ≥ 21, *I* = 18 − 20, *R* ≤ 17), ciprofloxacin (*S* ≥ 26, *I* = 22 − 25, *R* ≤ 21), gentamicin (*S* ≥ 15, *I* = 13 − 14, *R* ≤ 12), imipenem (*S* ≥ 23, *I* = 20 − 22, *R* ≤ 19), piperacillin–tazobactam (*S* ≥ 25, *I* = 21 − 24, *R* ≤ 20), tobramycin (*S* ≥ 15, *I* = 13 − 14, *R* ≤ 12), and trimethoprim–sulfamethoxazole (*S* ≥ 16, *I* = 11 − 15, *R* ≤ 10). MICs of meropenem for both the Kp5 and ATCC 13883 strains were determined using a broth microdilution assay according to CLSI guidelines [21]. Meropenem was serially two‐fold diluted in rows of a 96‐well microtiter plate (0.25–128 *μ*g/mL) to obtain final concentrations of 0.125–64 *μ*g/mL following the addition of bacterial inoculum. Subsequently, 50 *μ*L of bacterial suspension, adjusted to 0.5 McFarland standard (~1.5 × 10^8^ CFU/mL) turbidity using a spectrophotometer (Pharmacia, Novaspec II, Biochrom, England) and diluted 1:100 with M9 medium, was added to the wells to achieve a final inoculum concentration of 5 × 10^5^ CFU/mL in a total volume of 100 *μ*L. The plate was incubated at 37°C for 18 h, and the turbidity of each well was measured at 600 nm using the microplate spectrophotometer (Anthos Biotech, Salzburg, Austria) to determine bacterial growth. The MIC was defined as the lowest meropenem concentration that resulted in no visible growth. For each strain, the experiment was repeated three times. According to CLSI guidelines, *K. pneumoniae* is classified as susceptible when the MIC is ≤ 1 *μ*g/mL, intermediate when the MIC is 2 *μ*g/mL, and resistant when the MIC is ≥ 4 *μ*g/mL.

### 2.3. Antibacterial Activity of Xylitol

The MIC of xylitol was determined using the broth microdilution assay [21], with some modifications: Xylitol was substituted for the antibiotic, and M9 minimal medium was used instead of Mueller–Hinton broth [22]. Xylitol was dissolved in M9 medium, filter‐sterilized, and stored at −20°C until use.

A fresh bacterial culture was grown in M9 medium at 37°C, and the turbidity was adjusted to 0.5 McFarland standard using M9 medium as the diluent. This bacterial suspension was diluted 1:100 in M9 medium before the test to obtain the working inoculum. Serial two‐fold dilutions of xylitol (final concentrations 0.12–4 M) were prepared in M9 medium and dispensed into the wells of a sterile 96‐well microtiter plate (50 *μ*L per well). An equal volume (50 *μ*L) of the working inoculum was added to each well, resulting in a final inoculum of 5 × 10^5^ CFU/mL in a total volume of 100 *μ*L. Wells containing M9 medium without bacteria were used as negative controls, and wells containing bacteria without xylitol were used as growth controls. The microplates were incubated at 37°C for 18 h without shaking. After incubation, the turbidity of each well was measured at 600 nm using a spectrophotometer to determine bacterial growth. All experiments were performed in triplicate.

### 2.4. Checkerboard Susceptibility Testing

A two‐dimensional checkerboard assay was used to evaluate the synergistic antibacterial effect of xylitol in combination with meropenem, based on the guidelines described by Leber [23] with minor modifications. The culture medium was M9, and the final concentrations of xylitol and meropenem ranged from 0.12 to 4 M and 0.007 to 8 *μ*g/mL, respectively. Two‐fold serial dilutions of meropenem were added horizontally across the rows of a 96‐well microtiter plate (50 *μ*L per well). In comparison, two‐fold serial dilutions of xylitol were added vertically down the columns (50 *μ*L per well), resulting in wells with a unique concentration combination of both agents. A fresh bacterial culture was grown in M9 medium at 37°C, and the turbidity was adjusted to 0.5 McFarland standard using M9 medium as the diluent. The suspension was then diluted 1:31, and 10 *μ*L of the diluted suspension was added to each well as the inoculum, resulting in a final concentration of 4 × 10^5^–5 × 10^5^ CFU/mL in a total volume of 110 *μ*L per well. The microplates were incubated at 37°C for 18 h without shaking. After incubation, the turbidity of each well was measured at 600 nm using a spectrophotometer to determine bacterial growth.

The fractional inhibitory concentration index (FICI) for each combination of agents was calculated as FICI = FIC_meropenem_ + FIC_xylitol_, where FIC_meropenem_ = (MIC of  meropenem  in  combination/MIC  of  meropenem  alone) and FIC_xylitol_  =  (MIC of xylitol in combination/MIC of xylitol alone). For interpretation, FICI ≤ 0.5 indicates synergism, 0.5 < FICI ≤ 1 indicates additivity, 1 < FICI ≤ 4 indicates indifference, and FICI > 4 indicates antagonism [24].

### 2.5. Biofilm Formation Assay

To evaluate the biofilm formation ability of the bacterial strains, the microtiter plate assay method was used [25], with a modification to the culture medium.

Briefly, 10 *μ*L of fresh bacterial cultures grown in M9 medium at 37°C, with turbidity adjusted to 0.1 at OD_600_, was inoculated into flat‐bottom wells of 96‐well microtiter plates containing 90 *μ*L of M9 medium. Negative control wells contained only M9 medium. Microtiter plates were incubated at 37°C for 18 h under static conditions. After incubation, planktonic cells were aspirated, and wells were washed three times with PBS, dried at 60°C for 1 h, and stained with 100 *μ*L of 2% (*w*/*v*) crystal violet (CV) solution for 15 min. To remove excess CV, wells were washed with PBS, and the bound dye was solubilized by adding 100 *μ*L of 33% (*v*/*v*) glacial acetic acid to each well. Absorbance at 595 nm was measured after 30 min using a spectrophotometer. The assay was performed in triplicate for each strain.

A cut‐off value (ODc) was determined based on the average absorbance of negative controls and their standard deviation. Based on their absorbance (OD value) relative to the cut‐off, the ability of the strains to form biofilms was classified into one of four groups: nonbiofilm former (OD ≤ ODc), weak biofilm former (ODc < OD ≤ 2 × ODc), moderate biofilm former (2 × ODc < OD ≤ 4 × ODc), and strong biofilm former (4 × ODc < OD).

### 2.6. Biofilm Inhibition Assay

To investigate the inhibitory effect of xylitol on biofilm formation by the Kp5 and ATCC 13883 strains and to determine the minimum biofilm inhibitory concentration (MBIC), the method described by Haney et al. [26] was employed with minor modifications, including the use of M9 medium.

Briefly, xylitol was two‐fold serially diluted in M9 medium, and 50 *μ*L of each dilution was dispensed into flat‐bottom wells of a 96‐well microtiter plate, followed by the addition of 50 *μ*L of a freshly prepared bacterial suspension grown in M9 medium at 37°C, with turbidity adjusted to OD_600_ of 0.02 using the spectrophotometer, resulting in a final volume of 100 *μ*L per well. Final xylitol concentrations ranged from 0.12 to 4 M in treated wells. Wells containing M9 medium without bacteria served as sterility controls, while wells containing M9 medium with bacterial suspension but no xylitol served as growth controls. Plates were incubated at 37°C for 18 h under static conditions. After incubation, planktonic cells were aspirated, wells were washed three times with PBS, and the metabolic activity of live cells in the biofilm was measured using an MTT assay. To determine the total biofilm biomass, the CV method was performed in parallel on a replica plate prepared under identical conditions, as described earlier. These assays were performed in triplicate for each strain.

To calculate the percentage of biofilm metabolism, the following formula was used: 100 × ((OD_570,treatment_ − OD_570,sterility control_)/(OD_570,growth control_ − OD_570,sterility control_)). To calculate the percentage of biofilm biomass, the following formula was used: 100 × ((OD_595,treatment_ − OD_595,sterility control_)/(OD_595,growth control_ − OD_595,sterility control_)).

### 2.7. Biofilm Eradication Assay

To study the effect of xylitol on the eradication of preformed biofilms by the Kp5 and ATCC 13883 strains, a modified method based on that of Haney et al. [26] was employed.

Initially, biofilms were established in flat‐bottom wells of 96‐well microtiter plates as described above. The planktonic cells were then aspirated from each well, and the biofilms were rinsed three times with PBS. Following this step, 100 *μ*L of xylitol dilutions and 100 *μ*L of M9 medium were added to the preformed biofilms in each well for treatment (final concentration of xylitol ranging from 0.12 to 4 M for each isolate), resulting in a total volume of 200 *μ*L. Wells containing M9 medium without bacteria served as sterility controls, while wells containing M9 medium with bacterial suspension but no xylitol served as growth controls. Following 18 h of incubation at 37°C, the metabolic activity of live cells within the biofilm and the total biofilm biomass were determined using the MTT assay and CV method, respectively. Subsequently, the percentages of biofilm metabolism and biomass were calculated based on the aforementioned formulas. These assays were performed in triplicate for each strain.

### 2.8. Expression Analysis of the *ompK36* Gene

Real‐time PCR was used to determine *ompK36* gene expression in the presence and absence of 0.5 M xylitol for both strains. For this purpose, bacteria were inoculated into M9 medium with an OD_600_ of 0.1 and incubated at 37°C for 18 h. Xylitol (dissolved in M9 medium) was then added to the cultures to a final concentration of 0.5 M, and the cultures were incubated for 2 h. Controls received only M9 medium and were incubated under the same conditions. Bacterial cells were then harvested by centrifugation, and total RNA was extracted using TRIzol reagent (TIANGEN Co., China), according to the manufacturer′s protocol. The RNA sample was treated with DNase I (CinnaGen Co., Iran) to remove genomic DNA contamination and then subjected to single‐strand cDNA synthesis using a cDNA synthesis kit (Yekta Tajhiz Azma Co., Iran).

Real‐time PCR assays to measure *ompK36* expression were performed on a DTprime real‐time PCR instrument (DNA Technologies, Russia) according to the manufacturer′s instructions. The housekeeping gene *rrsH* (encoding 16S rRNA) was used to normalize the real‐time PCR data. The oligonucleotide sequences for the primers were as follows: *ompK36* forward primer, 5 ^′^TTAAAGTACTGTCCCTCCTGG3 ^′^; *ompK36* reverse primer, 5 ^′^TCAGAGAAGTAGTGCAGACCGTCA3 ^′^; *rrsH* forward primer, 5 ^′^GACGATCCCTAGCTGGTCTG3 ^′^; and *rrsH* reverse primer, 5 ^′^GTGCAATATTCCCCACTGCT3 ^′^.

Triplicate reaction mixtures contained 1 *μ*L of cDNA, 0.5 *μ*M of each forward and reverse primer, and RealQ Plus 1X master mix Green (Ampliqon, Denmark), adjusted to a final volume of 14 *μ*L with water. Following an initial denaturation at 95°C for 15 min, DNA was amplified with 40 cycles of 95°C for 30 s, 60°C for 30 s, and 72°C for 30 s, followed by a final extension step at 72°C for 5 min. Real‐time PCR data were analyzed according to the 2^−*Δ* *Δ*CT^ method to determine relative gene expression.

### 2.9. Statistical Analyses

Statistical analysis was performed using GraphPad Prism Version 10 software (GraphPad Software, San Diego, CA, United States). A *p* value of < 0.05 was considered statistically significant for all analyses. One‐way ANOVA with Dunnett′s post hoc test was used to compare the control group with each treatment group in the biofilm inhibition and eradication assays. Student′s *t*‐test was performed to compare the control and treated groups in the real‐time PCR gene expression analysis.

## 3. Results

### 3.1. Bacterial Identification and MIC Results

The Kp5 strain was identified as *K. pneumoniae* by detecting the *khe* gene, a species‐specific marker. The *khe* chromosomal gene, which encodes a hemolysin, is highly conserved in the *K. pneumoniae* genome.

Antibiogram results revealed that the Kp5 strain was resistant to all tested antibiotics, thereby classifying it as multidrug‐resistant (Table 1). The MICs of meropenem were 16 *μ*g/mL for the Kp5 strain and 0.12 *μ*g/mL for the ATCC 13883 strain, highlighting significant meropenem resistance in Kp5. The MIC of xylitol was 4 M for both strains, suggesting a similar inhibitory effect regardless of antibiotic resistance.

**Table 1:** Antimicrobial susceptibility profile of the clinical strain determined by the disk diffusion method.

Strain	Antibiotic	Zone diameter (mm)	Interpretation
Kp5	Ceftazidime	≤ 17	Resistant
	Ciprofloxacin	≤ 21	Resistant
	Gentamicin	≤ 12	Resistant
	Imipenem	≤ 19	Resistant
	Piperacillin–tazobactam	≤ 20	Resistant
	Tobramycin	≤ 12	Resistant
	Trimethoprim–sulfamethoxazole	≤ 10	Resistant

### 3.2. Synergistic Activity of Xylitol and Meropenem

A checkerboard assay was conducted to evaluate the potential synergistic effect between xylitol and meropenem. The FICI value was calculated for all combinations that resulted in complete inhibition of bacterial growth. Several of these growth‐inhibitory combinations exhibited synergistic interactions (FICI ≤ 0.5), with FICI values ranging from 0.24 to 0.5 for both the Kp5 and ATCC 13883 strains. The remaining combinations showed additive interactions (0.5 < FICI ≤ 1), with FICI values ranging from 0.51 to 1 for both strains. No indifferent or antagonistic interactions were observed in this study. Table 2 summarizes the MICs of meropenem and xylitol for all synergistic and additive interactions, along with the corresponding FIC and FICI values.

**Table 2:** Interactions of meropenem and xylitol against *K. pneumoniae* strains. Combinations of meropenem and xylitol tested against *K. pneumoniae* strains that resulted in synergistic or additive effects are presented. MICs of meropenem and xylitol in these combinations, FICs, and FICI values are shown. For reference, the MICs of meropenem alone were 16 *μ*g/mL for the clinical strain Kp5 and 0.12 *μ*g/mL for the standard strain ATCC 13883, and the MIC of xylitol alone was 4 M for both strains (values are rounded to two decimal places).

Strain	Meropenem		Xylitol		FICI	Interpretation
	MIC (*μ*g/mL)	FIC	MIC (M)	FIC		
Kp5	0.5	0.03	1	0.25	0.28	Synergism
	1	0.06	1	0.25	0.31	Synergism
	2	0.12	1	0.25	0.37	Synergism
	2	0.12	0.5	0.12	0.24	Synergism
	4	0.25	1	0.25	0.5	Synergism
	4	0.25	0.5	0.12	0.37	Synergism
	0.25	0.01	2	0.5	0.51	Additive
	0.5	0.03	2	0.5	0.53	Additive
	1	0.06	2	0.5	0.56	Additive
	2	0.12	2	0.5	0.62	Additive
	4	0.25	2	0.5	0.75	Additive
	8	0.5	2	0.5	1	Additive
	8	0.5	1	0.25	0.75	Additive
	8	0.5	0.5	0.12	0.62	Additive
	8	0.5	0.25	0.06	0.56	Additive
	8	0.5	0.12	0.03	0.53	Additive

ATCC 13883	0.03	0.24	1	0.25	0.5	Synergism
	0.03	0.24	0.5	0.12	0.37	Synergism
	0.03	0.24	0.25	0.06	0.31	Synergism
	0.03	0.24	0.12	0.03	0.28	Synergism
	0.03	0.24	2	0.5	0.74	Additive
	0.06	0.48	2	0.5	0.98	Additive
	0.06	0.48	1	0.25	0.73	Additive
	0.06	0.48	0.5	0.12	0.6	Additive
	0.06	0.48	0.25	0.06	0.54	Additive
	0.06	0.48	0.12	0.03	0.51	Additive

### 3.3. Biofilm Inhibition and Eradication by Xylitol

The biofilm‐forming abilities of the Kp5 and ATCC 13883 strains were quantified using the CV method. As shown in Table 3, both strains were classified as strong biofilm formers, with OD values exceeding 4 × ODc. The ODc value was determined to be 0.07.

**Table 3:** Biofilm formation ability of *K. pneumoniae* strains determined by the crystal violet (CV) method.

Strain	OD_595_ (*m* *e* *a* *n* ± *S* *D*)	ODc	Biofilm formation classification^a^
Kp5	1.17 ± 0.09	0.07	Strong
ATCC 13883	0.65 ± 0.14	0.07	Strong

^a^Nonbiofilm former (OD ≤ ODc), weak biofilm former (ODc < OD ≤ 2 × ODc), moderate biofilm former (2 × ODc < OD ≤ 4 × ODc), and strong biofilm former (4 × ODc < OD).

**Figure 1:**
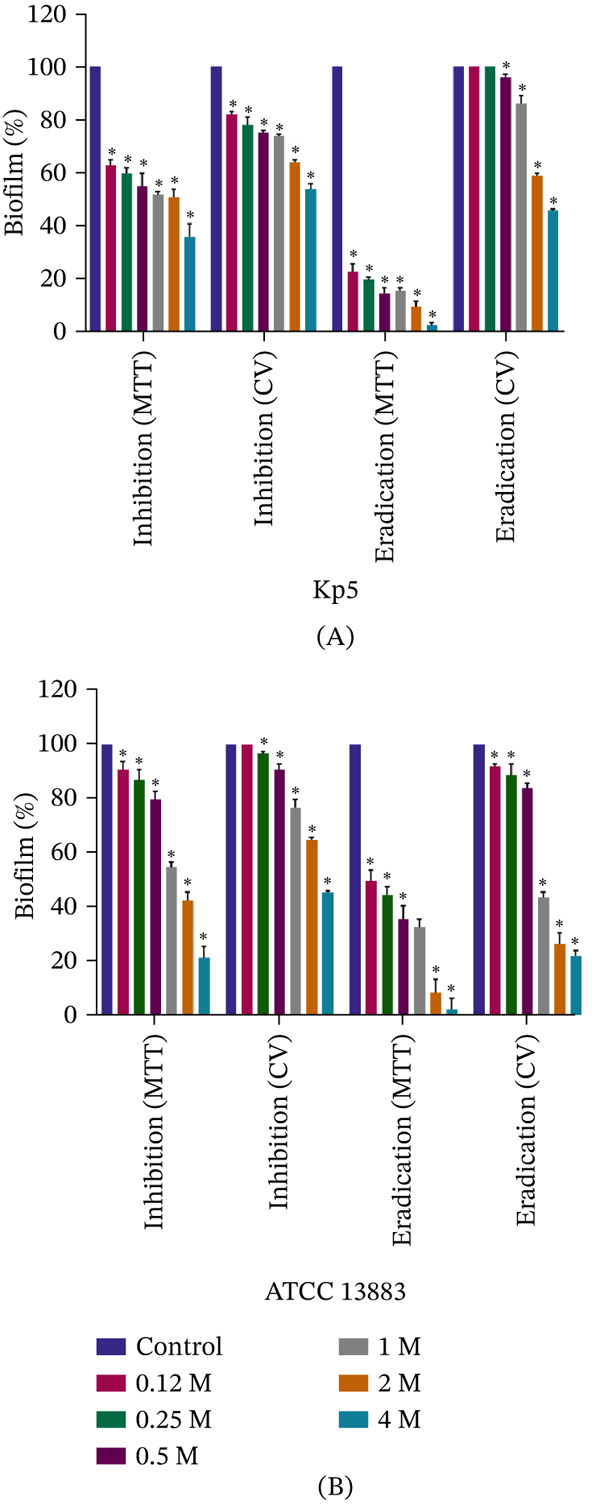
Effect of various concentrations of xylitol on biofilms of *K. pneumoniae* clinical strain Kp5 and standard strain ATCC 13883. Biofilm biomass was assessed using the CV method (inhibition and eradication assays), while biofilm activity was evaluated using the MTT assay (inhibition and eradication assays). (A) Kp5 and (B) ATCC 13883. Data are presented as mean ± SD. A one‐way ANOVA followed by Dunnett′s post hoc test was performed to compare each xylitol concentration to the untreated control group. Asterisks indicate a statistically significant difference from the untreated control group (*p* value < 0.05).

The effects of various xylitol concentrations (0.12–4 M) on biofilm inhibition, assessed using both the MTT assay and CV method, are presented in Figure 1. The MTT assay was employed to measure the metabolic activity of viable cells within the biofilm, thus reflecting the viability of biofilm‐embedded bacteria. In contrast, the CV method quantified total biofilm biomass, including bacterial cells and extracellular matrix. According to the results, xylitol exhibited a concentration‐dependent inhibitory effect on biofilm formation in both strains. For the Kp5 strain, at 0.12 M xylitol, biofilm biomass and metabolic activity were reduced to 82% and 63%, respectively, compared to the untreated group. At the highest tested xylitol concentration (4 M), these reductions were further extended to 54% for biofilm biomass and 36% for metabolic activity, compared to the untreated group. This inhibitory effect was statistically significant across all studied xylitol concentrations (0.12–4 M; p value < 0.05), as determined by one‐way ANOVA with Dunnett′s post hoc test. For the ATCC 13883 strain, biofilm biomass and metabolic activity were reduced to 46% and 22%, respectively, at 4 M xylitol, indicating greater susceptibility to this sugar alcohol compared with the Kp5 strain. In both strains, the MTT assay showed greater xylitol efficacy in inhibiting biofilm metabolic activity than the CV method, which measured total biofilm biomass.

The effects of xylitol concentrations (0.12–4 M) on biofilm eradication, assessed using both the MTT assay and CV method, are shown in Figure 1. For the Kp5 strain, the MTT assay demonstrated a more pronounced and significant effect of xylitol on biofilm eradication compared to the biofilm inhibition. Treatment with 4 M xylitol reduced biofilm metabolic activity to 3%, indicating a strong bactericidal effect within the mature biofilm. Even the lowest tested xylitol concentration (0.12 M) significantly reduced biofilm metabolic activity to 23%. In contrast, the CV method showed a lower xylitol eradication effect. While the two lowest concentrations (0.12 and 0.25 M) did not reduce biofilm biomass, treatment with 4 M xylitol reduced it to 46%, representing a significant but less pronounced reduction compared with the MTT assay results.

For the ATCC 13883 strain, xylitol also demonstrated a significant concentration‐dependent eradication effect. The MTT assay showed that treatment with 4 M xylitol reduced biofilm metabolic activity to less than 3%, similar to the effect observed in the Kp5 strain biofilms. The CV method showed a stronger biomass‐reducing effect in this strain compared with Kp5, with biofilm biomass reduced to 22% at 4 M xylitol. Notably, even the lowest xylitol concentration (0.12 M) significantly reduced biofilm biomass in the ATCC 13883 strain, again indicating greater susceptibility than in the Kp5 strain.

### 3.4. Real‐Time PCR Analysis

**Figure 2:**
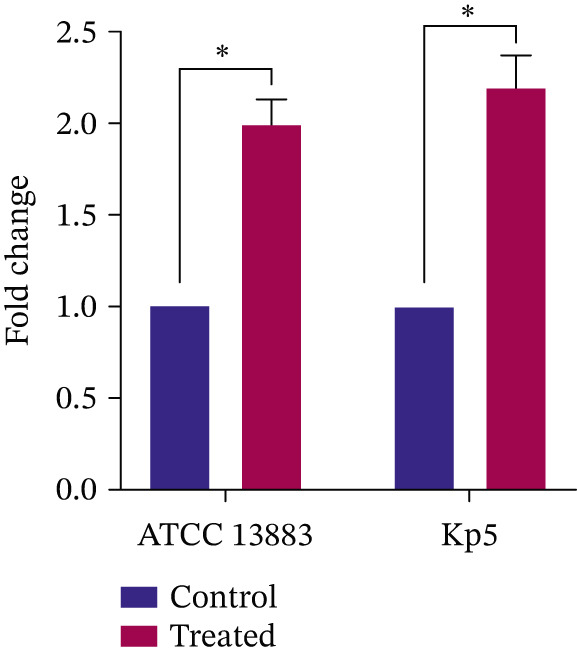
Relative gene expression of *ompK36* in *K. pneumoniae* clinical strain Kp5 and standard strain ATCC 13883. Expression was measured in the presence and absence of 0.5 M xylitol. Data are presented as mean ± SD. Statistical significance was determined using a Student′s *t*‐test. Asterisks indicate a statistically significant difference from the untreated control group (*p* value < 0.05).

The expression of the *ompk36* gene in the presence and absence of 0.5 M xylitol (the lowest xylitol concentration that resulted in synergism in both Kp5 and ATCC 13883 strains) was assessed by real‐time PCR in both strains. According to the results presented in Figure 2, the osmotic stress induced by 0.5 M xylitol increased the *ompk36* gene expression by 2.2‐fold in the Kp5 and 2.0‐fold in the ATCC 13883 strain. The upregulation of the *ompk36* gene was statistically significant in both strains, as determined by a Student′s *t*‐test.

## 4. Discussion

The World Health Organization (WHO) has identified carbapenem‐resistant Enterobacterales as a critical group of pathogens with limited treatment options and a high public health threat. Addressing the challenge caused by these pathogens requires investment in the development of new therapies and novel drugs, especially those that target OXA‐type *β*‐lactamases [27]. Following WHO recommendations, some studies have focused on finding novel peptides or small molecules to inhibit OXA‐type *β*‐lactamases and restore the carbapenem activities [28, 29]. Rather than inhibiting carbapenemases, this study assessed the synergy between xylitol and meropenem as a novel therapeutic approach to treat carbapenem‐resistant *K. pneumoniae*. Specifically, we evaluated whether high osmotic pressure induced by xylitol could increase *ompK36* expression, the gene encoding the OmpK36 porin, which is regulated by osmolarity and critical for carbapenem uptake, thereby increasing meropenem susceptibility. This was assessed in a clinical, meropenem‐resistant strain of *K. pneumoniae* (Kp5) and a standard strain, *K. pneumoniae* subsp. *pneumoniae* ATCC 13883.

Xylitol is a polyol with antibacterial effects, often attributed to its intracellular conversion into a toxic metabolite [17]; however, it has been shown that *K. pneumoniae* does not have the catabolic pathways to metabolize xylitol [30]. Consequently, xylitol cannot be used as a carbon source by this bacterium, nor can it be converted into a toxic molecule inside the cell. Therefore, the antibacterial and antibiofilm activity of xylitol observed in this study is likely due to the osmotic stress induced by this sugar alcohol or other unknown mechanisms. In contrast, several other common polyols, including glycerol, mannitol, galactitol, ribitol, and arabitol, are metabolized by *K. pneumoniae* and were thus unsuitable for use in this study [31–34].

Building on this premise, one of the critical mechanisms of carbapenem resistance in *K. pneumoniae* involves outer membrane porin levels. Various hydrophilic molecules, including nutrients and antibiotics, enter the cell via two major porins in this bacterium, OmpK35 and OmpK36, which are orthologs of OmpF and OmpC in *E. coli*, respectively. Specifically, the role of OmpK36 in carbapenem penetration into *K. pneumoniae* has been well documented, and studies have shown that the basic residues of this porin interact with the carboxylic groups of carbapenems. Accordingly, changes in porin expression or permeability are linked to antibiotic resistance in this bacterium [35]. The expression of OmpF and OmpC orthologs in Enterobacteriaceae is controlled by osmolarity, with high osmolarity conditions upregulating the expression of OmpC [36]. This regulatory mechanism has been the subject of various studies [37–39]. In one study evaluating the effect of osmotic stress on antibiotic resistance in *K. pneumoniae*, 2.5% NaCl (*w*/*v*) was added to the culture medium to increase the osmolarity. Real‐time PCR analyses revealed that under this osmotic stress, the expression of *ompK36* increased by about 1.7‐fold, which resulted in enhanced susceptibility to the antibiotics cefazolin and cefoxitin [6]. The results from the study reporting the influence of NaCl osmotic stress on the overexpression of *K. pneumoniae ompK36* are consistent with our results.

In this study, M9 medium was used to investigate the effect of xylitol. As a chemically defined medium, M9 allows more precise control of osmolarity and nutrient composition compared to complex media [40]. According to our results, osmotic stress induced by 0.5 M xylitol treatment for 2 h significantly upregulated *ompK36* expression in both the Kp5 and ATCC 13883 strains, by 2.2‐fold and 2.0‐fold, respectively, compared with untreated controls. This increased *ompK36* expression likely contributed to the observed reduction in meropenem MIC in both strains. Consistent with this result, synergistic activity between meropenem and xylitol was determined for both the Kp5 and ATCC 13883 strains. In the Kp5 strain, the meropenem MIC was reduced by up to 8‐fold in the presence of 0.5 M xylitol and by 32‐fold in the presence of 1 M xylitol, demonstrating synergistic activity (Table 2). For the ATCC 13883 strain, 0.12–1 M xylitol, when combined with meropenem, consistently reduced the meropenem MIC by 4‐fold (Table 2). The difference in the strains′ responses to the combination of xylitol and meropenem may be attributed to variations in membrane permeability. According to the CLSI interpretive criteria for meropenem (susceptible, ≤ 1 *μ*g/mL), xylitol treatment reduced the MIC of the meropenem‐resistant Kp5 strain to the intermediate resistance and susceptible range in synergistic combinations. These findings suggest that osmotic stress–induced upregulation of *ompK36* is likely the reason for the strains′ increased susceptibility in this study. Notably, the lowest concentration of xylitol resulted in synergism with meropenem for both strains in this study (0.5 M; 7.6% [*w*/*v*]), which is lower than concentrations typically used in commercial products, such as mouthwashes (up to 25%), nasal sprays (up to 35%), and chewing gums (66%–67%) [41].

In addition to its synergistic effect with meropenem, xylitol alone exhibited antibiofilm activity against both strong biofilm‐forming strains, Kp5 and ATCC 13883, by preventing biofilm formation and eradicating established biofilms in a concentration‐dependent manner. Biofilm eradication is a complex process that involves bacterial killing and/or biofilm dispersal. Because the CV staining method is nonspecific and stains all biological material, including the extracellular matrix, without distinguishing between live and dead cells, the metabolic activity of biofilm‐associated cells was assessed in parallel using the MTT assay to better reflect the viability of cells remaining within preformed biofilms [26]. For both strains, xylitol at concentrations of 0.25 M or higher significantly reduced biofilm formation, possibly by preventing cell attachment to surfaces and/or inhibiting metabolic activity. The antiadherent properties of xylitol against various Gram‐positive and Gram‐negative bacteria have been reported previously [42]. Moreover, our results suggest that xylitol′s efficacy in biofilm eradication is primarily due to inhibition of metabolic activity within the biofilm, with a lesser effect on total biofilm biomass. This is supported by the difference observed between the CV method (which measures biofilm biomass that may contain dead cells) and the MTT assay (which measures the metabolic activity of living cells). This difference shows that xylitol primarily inhibits bacterial metabolism in the biofilm and has a smaller effect on disrupting the biofilm matrix. Generally, xylitol had a greater effect on biofilm eradication in the ATCC 13883 strain than in the Kp5 strain, which may be attributed to strain‐specific differences in the biofilm matrix composition.

## 5. Conclusion

In this study, we demonstrated a novel therapeutic approach to combat carbapenem‐resistant *K. pneumoniae* by showing a synergistic effect between xylitol and meropenem. Our findings suggest that this synergy is likely mediated by xylitol‐induced osmotic stress, which upregulates the *ompK36* gene, thereby enhancing meropenem uptake into the bacterial cell. We also showed that xylitol alone exhibits significant antibiofilm activity, inhibiting biofilm formation and eradicating established biofilms, with its efficacy primarily attributed to its bactericidal activity against cells within the biofilm. These findings highlight xylitol′s dual potential as an effective stand‐alone antibiofilm agent and, more critically, as an adjuvant therapy to restore meropenem′s efficacy against multidrug‐resistant pathogens. This strategy of repurposing a safe, readily available compound to combat antimicrobial resistance offers a promising approach to addressing a major global public health crisis and warrants further investigation in in vivo studies. Additional research involving a larger collection of clinical isolates and other carbapenems, as well as the generation of a mutant strain in which the *ompK36* gene cannot be induced, is needed.

## Funding

This work was supported by Tehran University of Medical Sciences and Health Services (Grant No. 1403‐1‐101‐71647).

## Disclosure

The financial support received from Tehran University of Medical Sciences and Health Services was exclusively for the execution of the research. It did not involve the funder in the study design, data analysis, interpretation, or manuscript preparation.

## Conflicts of Interest

The authors declare no conflicts of interest.

## Data Availability

The datasets generated and/or analyzed during the current study are not publicly available due to being part of an ongoing project. Still, they are available from the corresponding author upon reasonable request.
